# Supplementary material for: Co-Occurrence of Mycotoxins in the Diet and in the Milk of Dairy Cows from the Southeast Region of Brazil
Source: Toxins (Basel). 2024 Nov 15;16(11):492. doi: 10.3390/toxins16110492 (PMC11598312; doi:10.3390/toxins16110492)
Supplement: Supplementary file 1 [file toxins-16-00492-s001.zip › toxins-3286160-Supplementary.pdf]

Supplementary Materials: Co-Occurrence of Mycotoxins in the Diet and in the Milk of Dairy Cows from the Southeast Region of Brazil

Aline Moreira Borowsky, Roice Eliana Rosim, Fernando Gustavo Tonin, Carlos Augusto Fernandes de Oliveira and Carlos Humberto Corassin

Table S1. Parameters used for mass spectrometry (MS) and tandem mass spectrometry (MS/MS) in conjunction with the methodology applied for the determination of mycotoxins in feed samples.

Mycotoxins	RT (min.)	Mass (g/mol)	Molecular ion	Transition (*m*/*z*)	LOD (μg/kg)	LOQ (μg/kg)
AFB_1	4,80	312,3	$[M+H]^+$	312,7 > 284,9 [a]	0,4	0,8
				312,7 > 241,1 [b]		
AFB_2	4,50	314,3	$[M+H]^+$	314,7 > 259,0 [a]	0,4	0,8
				314,7 > 287,0 [b]		
AFG_1	4,46	328,3	$[M+H]^+$	328,9 > 243,0 [a]	0,4	0,8
				328,9 > 199,5 [b]		
AFG_2	4,18	330,3	$[M+H]^+$	330,9 > 245,0 [a]	0,5	1,0
				330,9 > 188,9 [b]		
DON	1,98	296,3	$[M+H]^+$	397,3 > 249,1 [a]	6,1	18
				397,3 > 231,1 [b]		
OTA	5,99	403,1	$[M+H]^+$	404,0 > 238,9 [a]	0,5	1,0
				404,0 > 357,9 [b]		
FB_1	5,40	721,8	$[M+H]^+$	722,5 > 334,0 [a]	0,9	2,5
				722,5 > 352,1 [b]		
FB_2	3,74	705,8	$[M+H]^+$	706,5 > 336,2 [a]	0,7	2,0
				706,5 > 318,3 [b]		
T2	4,49	489,2	$[M+NH4]^+$	484,2 > 541,1 [a]	5,1	15
				484,2 > 542,0 [b]		
HT-2	4,30	447,2	$[M+NH4]^+$	442,2 >435,4 [a]	6,2	20
				442,2 >432,0 [b]		
ZEN	5,98	318.1	$[M-H]^-$	317,1 > 175,1 [a]	6,1	18
				317,1 > 130,9 [b]		

RT: Retention time; LOD: Limit of detection; LOQ: Limit of quantification; AF: aflatoxin; DON: deoxynivalenol; OTA: ochratoxin A; FB: fumonisin; T2: toxin T2; ZEN: zearalenone. [a]Transitions used in quantification. [b]Transitions used in confirmation.

Table S2. Parameters used for mass spectrometry (MS) and tandem mass spectrometry (MS/MS) in conjunction with the methodology applied for the determination of mycotoxins in milk samples.

Mycotoxins	RT (min.)	Mass (g/mol)	Molecular ion	Transition (*m/z*)	LOD (µg/kg)	LOQ (µg/kg)
AFM_1	4,09	328,3	$[M+H]^+$	329,0 > 273,1 [a]	0,005	0,015
				329,0 > 229,0 [b]		
DON	2,04	296,3	$[M+H]^+$	297,3 > 249,1 [a]	0,33	1,22
				297,3 > 231,1 [b]		
OTA	5,70	403,1	$[M+H]^+$	404,0 > 238,9 [a]	0,005	0,017
				404,0 > 357,9 [b]		
FB_1	5,34	721,8	$[M+H]^+$	722,5 > 334,0 [a]	0,30	0,62
				722,5 > 352,1 [b]		
FB_2	5,74	705,8	$[M+H]^+$	706,5 > 336,2 [a]	0,10	0,33
				706,5 > 318,3 [b]		
ZEN	6,01	318,1	$[M-H]^-$	317,1 > 175,1 [a]	0,10	0,30
				317,1 > 130,9 [b]		
α-ZEL	5,53	320,2	$[M-H]^-$	319,1 > 275,2 [a]	0,073	0,28
				319,1 > 160,2 [b]		
β-ZEL	5,76	320,2	$[M-H]^-$	319,1 > 275,2 [a]	0,060	0,20
				319,1 > 160,2 [b]		

RT: Retention time; LOD: Limit of detection; LOQ: Limit of quantification; AF: aflatoxin; DON: deoxynivalenol; OTA: ochratoxin A; FB: fumonisin; ZEN: zearalenone α-ZEL: α-zearalenol; β-ZEL: β-zearalenol. [a]Transitions used in quantification. [b]Transitions used in confirmation.
